# Podocyte Protein, Nephrin, Is a Substrate of Protein Tyrosine Phosphatase 1B

**DOI:** 10.1155/2011/376543

**Published:** 2011-10-15

**Authors:** Lamine Aoudjit, Ruihua Jiang, Tae Hoon Lee, Laura A. New, Nina Jones, Tomoko Takano

**Affiliations:** ^1^Division of Nephrology, Department of Medicine, McGill University, Montreal, QC, Canada H3A 2B4; ^2^Department of Molecular and Cellular Biology, University of Guelph, Guelph, ON, Canada NIG 2W1

## Abstract

Glomerular podocytes are critical for the barrier function of the glomerulus in the kidney and their dysfunction causes protein leakage into the urine (proteinuria). Nephrin is a key podocyte protein, which regulates the actin cytoskeleton via tyrosine phosphorylation of its cytoplasmic domain. Here we report that two protein tyrosine phosphatases, PTP1B and PTP-PEST negatively regulate nephrin tyrosine phosphorylation. PTP1B directly binds to and dephosphorylates nephrin, while the action of PTP-PEST is indirect. The two phosphatases are also upregulated in the glomerulus in the rat model of puromycin aminonucleoside nephrosis. Both overexpression and inhibition of PTP1B deranged the actin cytoskeleton in cultured mouse podocytes. Thus, protein tyrosine phosphatases may affect podocyte function via regulating nephrin tyrosine phosphorylation.

## 1. Introduction

**Figure 1:**
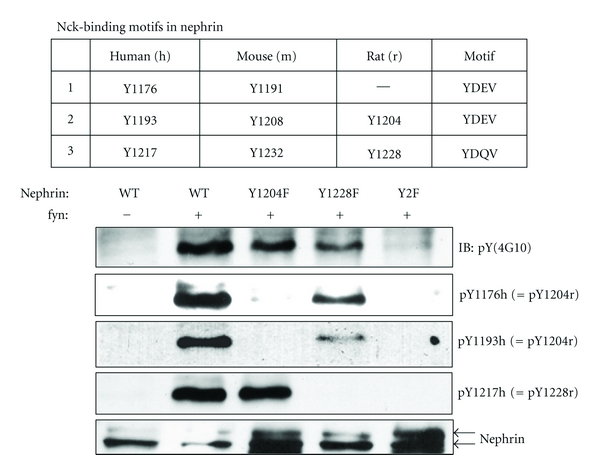
Nck-binding pY residues in human, mouse, and rat nephrin; validation of the phospho-specific antibodies with rat nephrin. In the cytoplasmic domain of rat nephrin, two tyrosine residues, Y1204 and Y1228, are responsible for Nck binding when phosphorylated [5]. The third Nck binding site (Y1176 in human and Y1191 in mouse) [2, 6] is absent in rat. Phospho-specific antibodies developed against the three Nck-binding sites in human [7] were tested against rat nephrin using Cos-1 cells transiently transfected with wild-type or mutant rat nephrin and fyn. pY1204r was recognized by both pY1176h and pY1193h antibodies, while pY1228r was recognized by only pY1217h antibody. Y2F: Y1204/1228F. Nephrin runs as a doublet (180/170 kDa) and p-nephrin corresponds to the upper band [3].

The glomerulus is the filtration unit of the kidney. When glomerular permselectivity (barrier function) is impaired, proteinuria occurs, which is a hallmark of many kidney diseases and a major contributing factor to the deterioration of kidney function. Visceral glomerular epithelial cells (commonly known as podocytes) are critical for structural integrity as well as permselectivity of the glomerulus. Nephrin (encoded by *NPHS1*) is a transmembrane protein expressed in podocytes and its mutations cause severe congenital nephrotic syndrome and renal failure known as Finnish type nephrotic syndrome [1]. The cytoplasmic domain of human nephrin contains 10 tyrosine (Y) residues, of which 7 are conserved in mouse and rat [2]. Some of these tyrosine residues are phosphorylated by Src-family kinases [3, 4]. Phosphorylation of human Y1176, 1193, and 1217 results in the recruitment of the adaptor protein, Nck, leading to a localized actin polymerization. The corresponding residues have the same role in mouse and rat, except that Y1176 is missing in rat (Figure 1) [2, 5, 6]. We have previously developed phospho-specific antibodies for the Nck-binding phosphotyrosine (pY) residues in human nephrin [7], which we also validated for rat (Figure 1). Actin polymerization induced by nephrin-Nck interaction leads to cellular process formation and morphological changes of the cell [5]. We [5, 8] and others [9] showed that nephrin tyrosine phosphorylation is significantly reduced in puromycin aminonucleoside nephrosis (PAN), a rat model of human minimal change disease and focal segmental glomerulosclerosis. Decreased nephrin tyrosine phosphorylation was accompanied by a decrease in F-actin in glomeruli [9]. More interestingly, Uchida et al. reported recently that phosphorylation of the two Nck binding sites (Y1193 and Y1217) is decreased in minimal change disease in humans [9]. Taken together, nephrin protein complex is a signaling nexus in podocytes wherein nephrin tyrosine phosphorylation appears to play a significant role in regulating actin cytoskeleton dynamics. However, little is known about how nephrin tyrosine phosphorylation is regulated. In particular, it is entirely unknown which phosphatase(s) is/are responsible for nephrin dephosphorylation.

Protein tyrosine phosphatases (PTPs) are defined by the active-site signature motif HCX_5_R. After the completion of the sequence of the human genome, approximately 100 PTPs are recognized in humans, of which 37 are known as classical phosphotyrosine-specific PTP [10]. There is a paucity of data regarding the expression and role of PTP in podocytes. The only well-characterized PTP that is expressed highly in podocytes is GLEPP1 [11, 12]. GLEPP1 is a transmembrane PTP which localizes in the apical membrane of podocytes (facing the Bowman's capsule), and GLEPP1 knockout mice display abnormal podocyte morphology and increased blood pressure after uninephrectomy [13]. Glombase analysis (large-scale gene identification in human glomerulus [14]) reveals 49 genes enriched in podocytes, which include only one PTP (GLEPP1). A separate study investigated the expression profile of PTP by RT-PCR in cultured mouse podocytes and demonstrated the expression of SHP2, PTP1B, PTP-PEST, and PTPD2 (formally known as PTP36) but not SHP1 or PTP20 [15]. Of interest, in this report, it was demonstrated that the nonspecific PTP inhibitor, sodium vanadate, causes dramatic morphological changes of cultured mouse podocytes, suggesting that PTP may have an important role in the cytoskeletal regulation of podocytes [15]. Since the expression of PTP in podocytes has not been studied systematically, it is possible that other PTP may also have a role in podocyte function.

The aim of the current study was to identify PTPs which dephosphorylate nephrin. Since GLEPP1 is localized in the apical membrane of podocytes, while nephrin is in the slit diaphragm close to the basolateral side, we focused our initial effort on other PTP, namely PTP1B, PTP-PEST, and SHP2, whose mRNA expression was previously shown in cultured podocytes [15].

## 2. Materials and Methods

### 2.1. Materials

pEBG-PTP1B-WT and D181A [16], pEBG-PTP-PEST-WT, and C231S [17] were from Dr. Tremblay (McGill University). Wild-type PTP1B, which is tagged with green fluorescent protein (GFP-PTP1B-WT), was from Dr. Arregui (Universidad de San Martín, Buenos Aires, Argentina) [18]. Fyn and rat nephrin plasmids were described previously [3]. Antibodies were purchased from the following sources: PTP1B and SHP2 (BD Biosciences), PTP-PEST (Cell Signaling), *α*-tubulin (Abcam), 4G10 (for pY, Millipore), GST and fyn (Santa Cruz Biotech.), pY418-Src (Invitrogen). Antinephrin antibody [3] and phospho-specific nephrin antibodies [7] were described previously. PTP1B inhibitor was from Calbiochem (#539741: 3-(3,5-dibromo-4-hydroxy-benzoyl)-2-ethyl-benzofuran-6-sulfonicacid-(4-(thiazol-2-ylsulfamyl)-phenyl)-amide).

### 2.2. Cells

Immortalized cultured mouse podocytes stably expressing rat nephrin were described previously [19]. These cells were maintained in permissive conditions at 33°C and were differentiated by a temperature switch to 37°C for 7–14 days [19]. Cos-1 cells were cultured in DMEM-10% FBS. Transient transfection and immunofluorescence staining were carried out as described previously [19].

### 2.3. GST Pulldown

Cos-1 cells were transfected with pEBG-PTP1B or pEBG-PTP-PEST (encoding GST-tagged PTP) with nephrin. On the following day, cells were lysed in lysis buffer (25 mM HEPES (pH 7.5), 1% TritonX-100, 10 mM MgCl_2_, 100 mM NaCl, 5% glycerol, 5 mM NaF, 1 mM PMSF, 10 *μ*g/mL aprotinin, 10 *μ*g/mL leupeptin). Equal amounts of protein (250 *μ*g) were incubated for 2 h at 4°C with glutathione-sepharose beads. The beads were washed twice and were subjected to SDS-PAGE and immunoblotting. 

### 2.4. qPCR

Total RNA was prepared from glomeruli using Trizol Reagent (Invitrogen). cDNA synthesis was performed using a QuantiTect Reverse Transcription kit (Qiagen, Mississauga, ON). The PCR primer sets used were as follows: PTP1B fwd: 5′-ggaactgggcggctatttacc-3′, rev: 5′- caaaagggctgacatctcggt-3′; PTP-PEST fwd: 5′-gtcgagaatttgagatgggaagg-3′, rev: 5-gtcgagaatttgagatgggaagg-3′. qPCR was carried out with iTaq SYBR Green supermix with ROX (Biorad) and an ABI 7300 System (Applied Biosystems) using the delta Ct method with GAPDH as an endogenous control.

### 2.5. Puromycin Aminonucleoside Nephrosis (PAN) and Isolation of Rat Glomeruli

PAN was induced with a single intravenous injection of puromycin aminonucleoside (Sigma-Aldrich Canada, Mississauga, ON, 50 mg per kg) in male Sprague-Dawley rats (150–175 g), as previously described [8]. Isolation of rat glomeruli was performed by differential sieving and lysates were prepared in buffer containing 1% Triton X-100, as described previously [8]. Studies were approved by the Animal Care Committee at McGill University.

### 2.6. Immunohistochemistry

Immunohistochemistry of rat kidney sections was performed as described previously [20]. Primary antibodies used were rabbit anti-PTP-PEST antiserum (gift from Dr. Michael Tremblay, McGill University, 1 : 100) [21] and rabbit anti-PTP1B monoclonal antibody (Epitomics, 1 : 50).

### 2.7. Statistics

Data are presented as mean ± SEM. One-way analysis of variance (ANOVA) was used to determine significant differences among groups and where significant differences were found, individual comparisons were made between groups using the *t* statistic and adjusting the critical value according to the Bonferroni method.

## 3. Results and Discussion

### 3.1. PTP1B, PTP-PEST, and SHP2 Are Expressed in Cultured Mouse Podocytes

**Figure 2:**
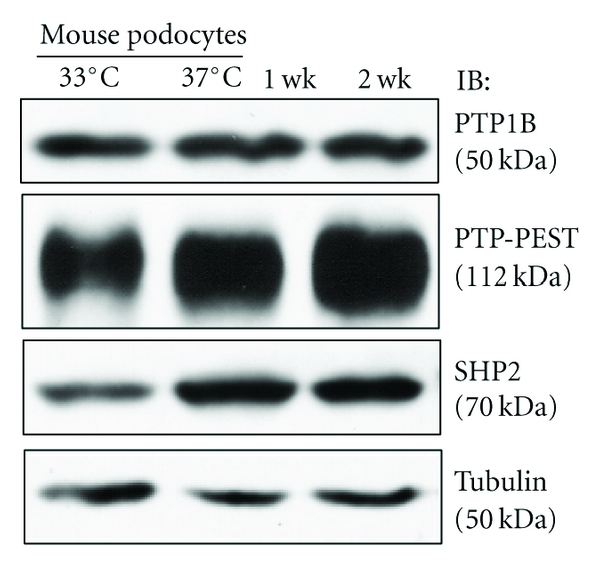
PTP1B, PTP-PEST, and SHP2 are expressed in cultured mouse podocytes. Immortalized mouse podocytes were either un-differentiated or differentiated for 1-2 wks and cell lysates were analyzed by immunoblotting to confirm the expression of the three PTP.

We first studied the expression of the three well-studied classical PTPs, namely, PTP1B, PTP-PEST, and SHP2 in cultured mouse podocytes. By immunoblotting, these three PTPs were clearly expressed in both un-differentiated and differentiated mouse podocytes at the protein level, consistent with the previous report which showed mRNA expression of these PTP by RT-PCR (Figure 2). Glomerular expression of the three PTPs has also been demonstrated by immunohistochemistry in normal human kidney (Human Protein Atlas, http://www.proteinatlas.org/).

### 3.2. PTP1B and PTP-PEST, but Not SHP2, Inhibit Fyn-Induced Nephrin Tyrosine Phosphorylation

**Figure 3:**
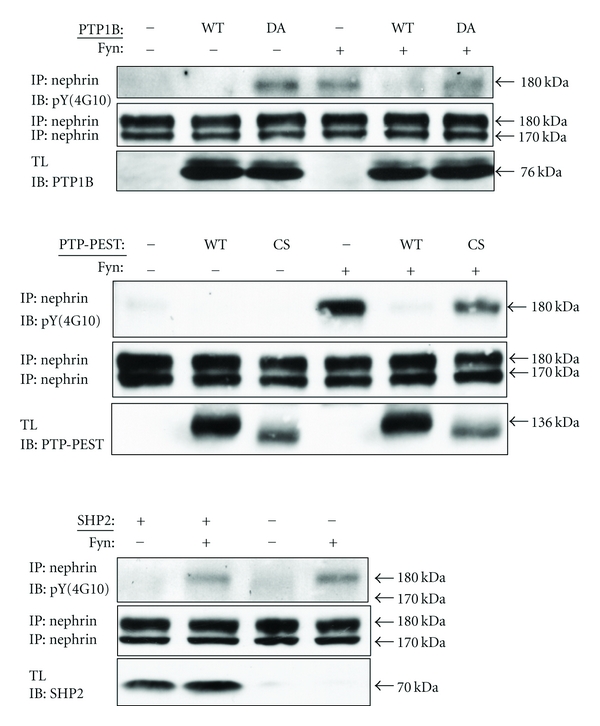
PTP1B and PTP-PEST, but not SHP2, inhibit fyn-induced nephrin phosphorylation. Cos-1 cells were transiently transfected with the indicated plasmids and cell lysates were analyzed after 24 hrs by immunoprecipitation/immunoblotting. When expressed alone, tyrosine phosphorylation of nephrin was weak, which was markedly increased by fyn. PTP1B and PTP-PEST inhibited fyn-induced tyrosine phosphorylation of nephrin, while SHP2 did not have any impact. TL: total lysates. PTP1B and PEP-PEST were tagged with GST. WT indicates wild type. DA and CS indicate substrate trapping (catalytically inactive) mutants of PTP1B and PTP-PEST, respectively (see Section 2).

We and others showed previously that the cytoplasmic domain of nephrin is tyrosine phosphorylated by Src-family kinases, preferentially by fyn [3, 4]. We thus studied whether the three PTPs affect fyn-induced nephrin phosphorylation. When nephrin alone was transiently expressed in Cos-1 cells, its tyrosine phosphorylation was barely detectable by immunoblotting, which was markedly increased by coexpression of fyn (Figure 3). Fyn-induced phosphorylation of nephrin was almost completely abolished by co-expressing PTP1B or PTP-PEST but was unaffected by SHP2 or catalytically inactive mutants of PTP1B and PTP-PEST (Figure 3). Thus, PTP1B and PTP-PEST, but not SHP2, are likely to contribute to dephosphorylation of nephrin.

### 3.3. PTP1B Directly Binds to and Dephosphorylates Nephrin

**Figure 4:**
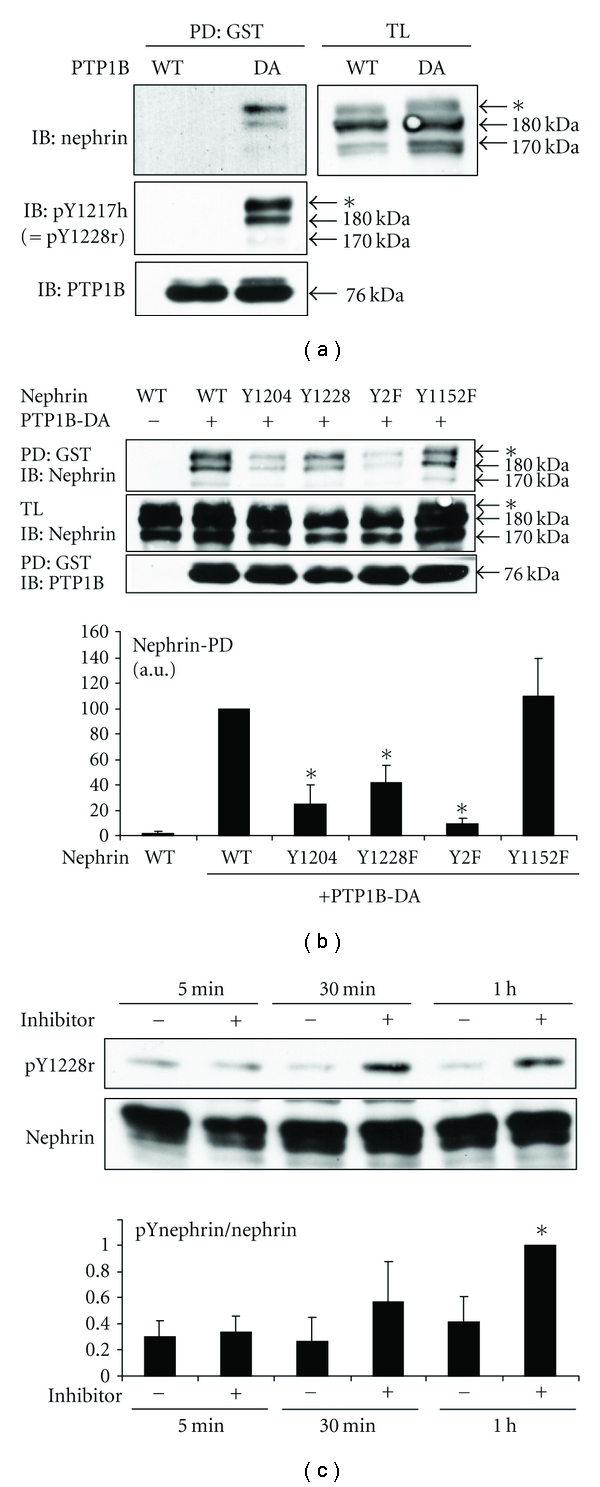
PTP1B binds to and dephosphorylates nephrin. (a) Cos-1 cells were transiently transfected with nephrin and GST-PTP1B-WT or GST-PTP1B-DA (substrate-trapping mutant). Cell lysates were subjected to pull-down (PD) with glutathione beads and analyzed by immunoblotting. PTP1B-DA, but not WT, pulled down nephrin while preserving its phosphorylation. Nephrin pulled down by PTP1B-DA appeared as a doublet and both bands were phosphorylated—the bottom band corresponded to nephrin at 180 kDa, while the top band (indicated by *) ran above 180 kDa, possibly representing a hyperphosphorylated form. (b) Pull-down by PTP1B-DA was performed using Y-to-F mutants of nephrin. Mutation of the two Nck binding sites in rat (Y1204 and Y1228) [5] additively affected nephrin-PTP1B interaction, while mutation of another Y residue (Y1152, phosphoinositide-3 kinase binding site) [8] had no effect. Densitometric analysis, normalized to total nephrin, is shown. *P < 0.05 versus WT, *n* = 4. (c) Undifferentiated cultured mouse podocytes were serum starved in 0.5% FBS overnight and were incubated with the PTP1B inhibitor (50 *μ*M) or vehicle for the indicated times. Cell lysates were analyzed by immunoblotting for pY1217h (=pY1228r)-nephrin and total nephrin. Bottom: densitometric analysis is shown. Results were normalized to total nephrin. *P < 0.05 versus no inhibitor, *n* = 4.

We next asked if PTP1B dephosphorylates nephrin directly. The D181A mutant of PTP1B (PTP1B-DA) retains the ability to bind to pY containing substrates but lacks the phosphatase activity and is called “substrate-trapping mutant” [16]. A substrate-trapping mutant can form stable complex with the substrates and can be used to pull down bound substrates. Since pY residues are protected from endogenous phosphatases by substrate trapping mutants, substrates are typically hyperphosphorylated in the presence of substrate trapping mutants. When transfected in Cos-1 cells, PTP1B-DA pulled down nephrin effectively, while wild-type PTP1B did not (Figure 4(a)). Nephrin, which was pulled down by PTP1B-DA was strongly phosphorylated, consistent with the ability of the substrate-trapping mutants to bind to and protect pY from endogenous phosphatases (Figure 4(a)). The ability of PTP1B-DA to pull down nephrin was markedly impaired when the Nck-binding tyrosine residues, Y1204 or Y1228 in rat, were mutated to phenylalanine and was virtually abolished when both residues were mutated simultaneously (Figure 4(b)). In contrast, mutation of the phosphoinositide-3 kinase binding site, Y1152 (rat), did not affect nephrin-PTP1B-DA interaction (Figure 4(b)). Finally, when mouse podocytes stably overexpressing rat nephrin were incubated with a specific PTP1B inhibitor for one hour, phosphorylation of Y1228 (rat) was augmented significantly (Figure 4(c)). Taken together, we conclude that PTP1B directly binds to and dephosphorylates the Nck-binding sites (pY1204 and pY1228) of rat nephrin.

### 3.4. PTP-PEST Decreases Nephrin Phosphorylation Indirectly

**Figure 5:**
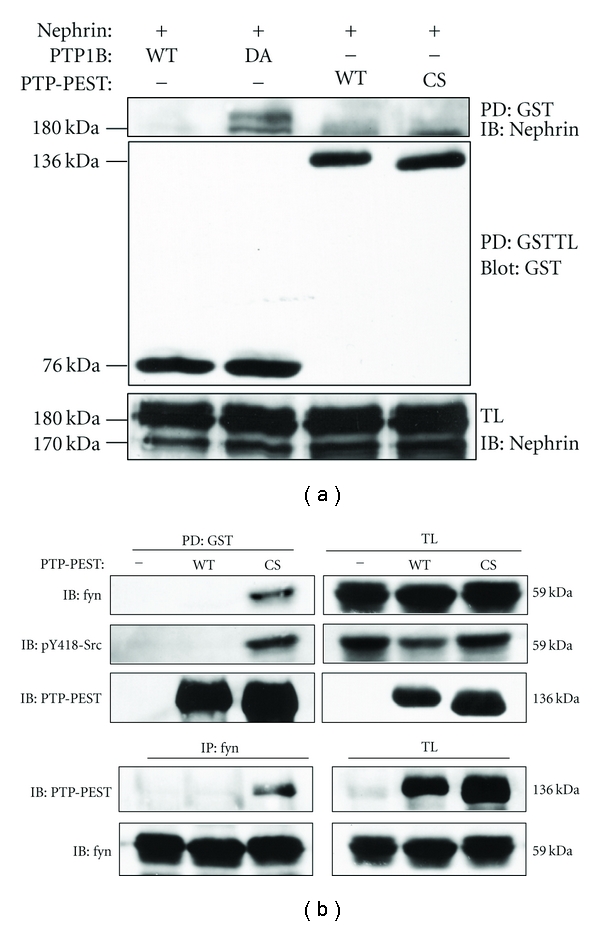
PTP-PEST decreases nephrin phosphorylation via Src-family kinase. (a) Pull-down was performed as in Figure 4(a) with additional plasmids of GST-PTP-PEST-WT and GST-PTP-PEST-CS (substrate-trapping mutant). The substrate-trapping mutant of PTP1B, but not of PTP-PEST, pulled down nephrin. (b) Cos-1 cells were transiently cotransfected with pEBG (empty vector), GST-PTP-PEST-WT, or GST-PTP-PEST-CS (substrate trapping mutant) and fyn. After 24 hrs, cell lysates were subjected to GST pull down as in Figure 4(a) or to immunoprecipitation for fyn. Only the substrate-trapping mutant of PTP-PEST (CS), but not the wild-type, pulled down fyn or was coimmunoprecipitated with fyn. fyn, which was pulled down by PTP-PEST-CS, was strongly phosphorylated at pY418, suggesting that pY418 containing region is the substrate of PTP-PEST. Wild-type PTP-PEST decreased pY418-Src in total lysates. Note that pY418 antibody cross-reacts to all the Src-family kinases.

We next studied nephrin-PTP-PEST interaction using a similar strategy. In contrast to PTP1B, the substrate-trapping mutant of PTP-PEST, PTP-PEST-C231S [17], failed to pull down nephrin in Cos-1 cells, indicating that nephrin is not a direct substrate of PTP-PEST (Figure 5). It was reported previously that the activity of the Src-family kinases could be regulated by PTP [22–24]. Thus, we next studied if fyn phosphorylation and activity were affected by PTP-PEST. It is established that phosphorylation at Y418 in the catalytic site is critical for the activation of Src-family kinases, and thus, is used as a marker of activation [25]. When transfected in Cos-1 cells, PTP-PEST (wild type) decreased the phosphorylation of Y418 (Figure 5(b)-right, 2nd panel). The substrate trapping mutant, PTP-PEST-CS, but not the wild type, pulled down fyn (Figure 5(b)-left, 1st panel) which was strongly phosphorylated onY418 (Figure 5(b)-left, 2nd panel). Similarly, the substrate trapping mutant of PTP-PEST but not the wild type coimmunoprecipitated with fyn (Figure 5(b)-lower panels). These results suggest that PTP-PEST binds to and dephosphorylates fyn at Y418, thereby inhibiting its activity. Inhibition of fyn in turn is likely to contribute to reduced phosphorylation of nephrin.

### 3.5. Both Overexpression and Inhibition of PTP1B Cause Deranged Actin Cytoskeleton and Cell Morphology in Podocytes

**Figure 6:**
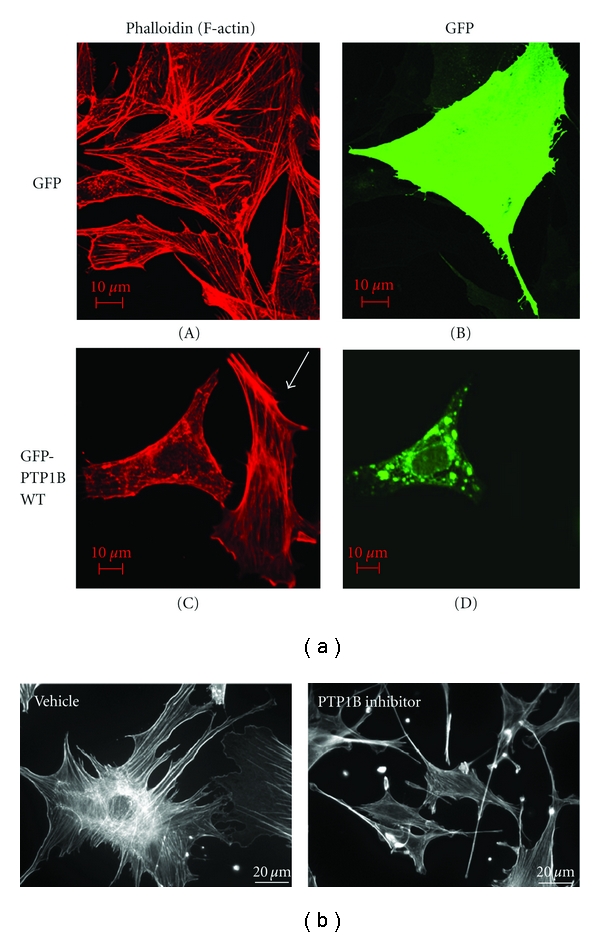
Both overexpression and inhibition of PTP1B cause deranged actin cytoskeleton and cell morphology in podocytes. (a) Mouse podocytes were differentiated for 10 days and were transfected with GFP alone or GFP-PTP1B-WT. On the following day, cells were fixed, permeabilised, and stained with phalloidin to visualize F-actin. GFP transfected cells (A) or untransfected cells (C, arrow) showed well-defined stress fibers, as reported previously. Cells transfected with GFP-PTP1B-WT (C, left) showed less stress fibers and fine aggregates of F-actin. (b) Mouse podocytes were differentiated for 10 days in the presence or absence of the PTP1B inhibitor (50 *μ*M, IC50: 4–8 *μ*M, see Section 2). The inhibitor caused marked morphological changes with smaller cell size, collapsed appearance of cellular processes, and elongated cell shape, although the stress fibers were well preserved.

In order to address the function of PTP1B in podocytes, we next overexpressed wild-type PTP1B tagged with GFP in differentiated mouse podocytes (Figure 6(a)). GFP transfected cells (Figure 6(a)-(A)) or untransfected cells (Figure 6(a)-(C), arrow) showed well-defined stress fibers, as reported previously [19]. By contrast, GFP-PTP1B-WT showed granular distribution, similar to the previous report [18]. Cells transfected with GFP-PTP1B-WT (Figure 6(a)-(C), left) showed less stress fibers and fine aggregates of F-actin. We next examined differentiated mouse podocytes cultured in the presence or absence of the PTP1B inhibitor (Figure 6(b)). The inhibitor caused marked morphological changes with smaller cell size, collapsed appearance of cellular processes, and elongated cell shape, although the stress fibers were well preserved. These results suggest that the appropriate amount of PTP1B is necessary to maintain normal cell structure of podocytes.

### 3.6. Glomerular PTP1B and PTP-PEST Are Upregulated in the Rat Model of Proteinuria

**Figure 7:**
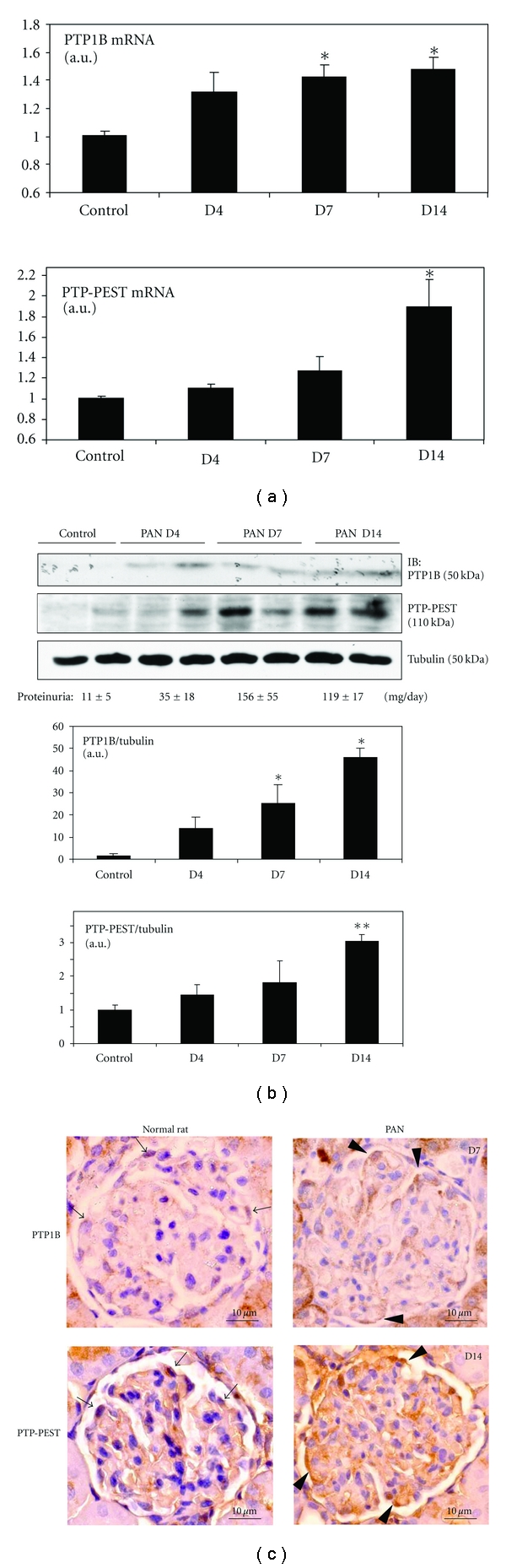
PTP1B and PTP-PEST are upregulated in glomeruli of rats with PAN. PAN was induced and glomeruli were isolated by differential sieving as in Section 2. (a) mRNA expression of *PTP1B* and *PTP-PEST* was quantified by qPCR. *P < 0.05 versus control, *n* = 8 rats for PTP1B and *n* = 6 rats for PTP-PEST. (b) Glomerular lysates were analyzed in duplicate by immunoblotting. Both PTP1B and PTP-PEST were upregulated in a similar time course to the respective mRNAs. Values for proteinuria are mean ± SEM of 4 rats. Lower panel: densitometric analysis for PTP1B (*N* = 6 rats) and PTP-PEST (*N* = 4 rats) proteins. *P < 0.05, **P < 0.01 versus control (c) Immunohistochemistry of control and PAN-treated rat kidneys. In normal rat glomeruli, weak PTP1B and PTP-PEST expression was detected with some staining in the extracapillary location compatible with podocytes (arrows). In the kidney from PAN rats, expression of the two PTP was upregulated, in particular in podocytes (arrowheads, day 7 for PTP1B and day 14 for PTP-PEST). There was no staining when the primary antibodies were replaced with control IgG (not shown).

Puromycin aminonucleoside nephrosis (PAN) is a rat model of podocyte injury and proteinuria and is widely accepted as a model of minimal change disease or focal segmental glomerulosclerosis in humans [26]. We and others reported previously that tyrosine phosphorylation of nephrin at the Nck-binding sites, that is, Y1204 and Y1228 in rat is decreased significantly in this model [7–9]. Since PTP1B and PTP-PEST decreased nephrin phosphorylation *in vitro*, we next studied the expression level of these two PTPs in glomeruli of rats with PAN. In this model of podocyte injury, proteinuria typically starts on day 4, peaks on day 7, and starts to decline on day 14 after disease induction, while decrease of nephrin tyrosine phosphorylation is observed starting on day 4 and persisting until day 14 [7]. By qPCR, *PTP1B* mRNA was significantly upregulated on days 7 and 14, while upregulation of *PTP-PEST* mRNA was significant only on day 14 (Figure 7(a)). Immunoblotting showed protein upregulation of these two PTP in the time course similar to respective mRNAs (Figure 7(b)). Furthermore, increased expression of PTP1B and PTP-PEST could also be seen in podocytes of kidney sections obtained from PAN-treated rats when compared to normal control rats (Figure 7(c)). Upregulation of the proteins were more distinct, as compared with that of mRNAs, suggesting that there may be posttranslational modification, in addition to transcriptional or posttranscriptional regulations of the mRNAs (Figure 7(b)). Although causal relationship cannot be established, these results are consistent with the notion that upregulation of PTP1B and PTP-PEST contributes to decreased tyrosine phosphorylation of nephrin, which may in turn result in impaired cytoskeletal regulation in podocytes and development of proteinuria.
